# Novel developments in computational spectropolarimeter

**DOI:** 10.1038/s41377-023-01097-3

**Published:** 2023-03-01

**Authors:** En-Lin Hsiang, Shin-Tson Wu

**Affiliations:** College of Optics and Photonics, University of Central Florida, Orlando, FL 32816 USA

**Keywords:** Optics and photonics, Physics

## Abstract

Compared to conventional bulky spectropolarimeters, computational spectropolarimeters which reconstruct light-field information such as polarization and spectrum in a compact form factor, are critical equipment enabling new applications. The key component of a computational spectropolarimeter is a tunable light-field modulator, in which liquid crystal-based device is a promising candidate. By varying the applied voltage, the tunable liquid crystal metasurface can modulate the phase and spectral information of the incident light, and after a few trials, this important information can be decoded mathematically. Such a novel approach paves the foundation for developing compact and low-cost spectropolarimetric imaging devices with widespread applications in biomedical imaging, remote sensing, and optical communications.

In contrast to photodetectors which can only measure light intensity, spectropolarimeter is able to measure light-field properties such as spectrum and polarization. As a result, spectropolarimetry is a fundamental device enabling new applications. For example, by revealing the polarization and spectrum of light passing through a target material, one can characterize the material’s composition, refractive index, and birefringence. In addition, polarimetry is also widely used in astronomy, remote sensing, radar and so on^1,2^. Overall speaking, spectropolarimeters are useful instruments in physics, astronomy, chemistry, and biology. Traditional spectropolarimetry spatially separates a light beam with different polarization by a polarizing beam splitter and different wavelength by a grating^3^. Afterward, a spatially 2D photodetector is used to measure the light intensity. The intensity on each position of the photodetector corresponds to a different polarization state or wavelength. Therefore, the polarization state and spectrum of light can be restructured by summarizing the spatial intensity distribution from the 2D photodetector. Because a 2D detector array is required for measuring the polarization and spectrum at a single spatial location, its use for spectropolarimetric imaging is deterred. Meanwhile, the polarization and spectrum of a light beam can also be obtained by the sequential method, in which the polarization and wavelength filters are sequentially applied to measure the light intensity with different polarization and wavelength components^4^. Although the above-mentioned spectropolarimeters have been widely used, the sophisticated optical system and bulky form factor limit their application to some miniaturized platforms.

Recently, a computational spectropolarimeter has been proposed, whose working principle is illustrated in Fig. 1a^5^. During this design process, the incident light with unknown optical field properties is modulated (encoded) by a tunable metasurface and then received by a photodiode. In each trial, the tunable metasurface provides a different phase and different spectral reflectance to encode the incident light. Then, with multiple trials, the polarization and spectrum of the incident light can be decoded by computational reconstruction. In 2018, Jung et al.^6^ developed a mid-infrared polarimetry device using a graphene-integrated anisotropic metasurface. Although the accuracy of polarization measurements is degraded by the depolarization effects of sample defects, a measurement rate up to tens of MHz can be achieved, which is about 100× faster than that of conventional mechanical rotation of polarizer/retarder modulators.

**Fig. 1:**
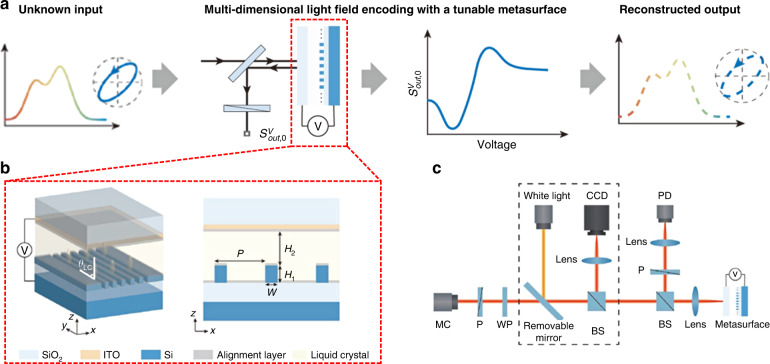
Computational spectropolarimetry with a tunable liquid crystal metasurface. **a** Illustration of computational spectropolarimeter’s working principle. **b** Schematic of electrically tunable liquid crystal-embedded silicon metasurface. **c** Experimental setup for the spectropolarimetric measurements

Liquid crystal materials with anisotropic optical properties have been widely used in displays and optical devices to modulate the amplitude and phase of light^7,8^. In this work, Ni et al.^5^ demonstrated a method to modulate the wavelength and polarization information of light using an electrically tunable liquid crystal-embedded silicon metasurface as shown in Fig. 1b, which is tailored to support multi-guided mode resonance. By applying a series of different voltages, the liquid crystal metasurface encodes the incoming light, which is then recorded by a single-pixel photodetector. Afterward, the polarization and spectrum of the incident light can be reconstructed by computational reconstruction algorithms. To accurately reconstruct the polarization and spectrum of light, it takes about 20–30 iterations. Considering the response time of liquid crystal devices, which is usually several milliseconds, the total measurement time amounts to tens of milliseconds, corresponding to ~100 Hz frame rate. The total measurement time can be reduced by choosing a fast-switching liquid crystal and advanced reconstruction algorithms. Furthermore, in practice, the orientation of liquid crystal directors may not be so uniform as illustrated in the simulation model. The anchoring energy, liquid crystal material properties, and applied electric field all affect the alignment uniformity under different driving conditions. For example, the liquid crystal directors nearby the alignment layer with strong anchoring energy will be more difficult to be reoriented by the applied electric field^9^. This uneven liquid crystal alignment adds noise to the tailored reflectance spectrum and phase modulation during the encoding process. Therefore, further optimization of liquid crystal devices is required. Despite the above-mentioned issues, this new spectropolarimeter which can measure the polarization and spectrum of light simultaneously with high fidelity paves the foundation for developing compact and low-cost spectropolarimeters. More importantly, as Fig. 1c depicts, a major advantage of this approach is that the liquid crystal metasurface does not change the wavefront of the incident light. Thus, by combining with a detector array, spectropolarimetric imaging can be realized, which could have significant implications for biomedical imaging, remote sensing, and optical communications.
